# *In Vitro* Interactions of Antifungal Agents and Everolimus Against *Aspergillus* Species

**DOI:** 10.3389/fcimb.2022.936814

**Published:** 2022-07-05

**Authors:** Huiping Jiang, Jianqun Xiong, Lihua Tan, Ping Jin, Yi Sun, Lianjuan Yang, Jingwen Tan

**Affiliations:** ^1^ Department of Critical Care Medicine, Jingzhou Central Hospital, The Second Clinical Medical College, Yangtze University, Jingzhou, China; ^2^ Department of Pharmacy, Jingzhou Central Hospital, The Second Clinical Medical College, Yangtze University, Jingzhou, China; ^3^ Department of Dermatology, Jingzhou Central Hospital, Candidate Branch of National Clinical Research Center for Skin and Immune Diseases, The Second Clinical Medical College, Yangtze University, Jingzhou, China; ^4^ Department of Medical Mycology, Shanghai Skin Disease Hospital, Tongji University School of Medicine, Shanghai, China

**Keywords:** TOR pathway, TOR inhibitor, everolimus, azoles, *Aspergillus*

## Abstract

Multiple cellular activities, including protein and lipid synthesis, ribosome biogenesis, and metabolic processes, are regulated by the target of rapamycin (TOR) pathway. Recent research suggests that the TOR might play an important role in various physiological functions of pathogenic fungi, such as nutrient sensing, stress response, and cell cycle progression. Given their robust immunosuppressant and antitumor activities, TOR inhibitors are widely used in clinical settings. In the present study, a microdilution checkerboard-based approach was employed to assess the interactions between the oral mammalian target of rapamycin (mTOR) inhibitor everolimus (EVL) and antifungal agents in the treatment of *Aspergillus* species derived from 35 clinical isolates *in vitro.* The results revealed that EVL exhibited promising inhibitory synergy with itraconazole (ITC), posaconazole (POS), and amphotericin B (AMB) for 85.7%, 74.2%, and 71.4%, respectively. In contrast, EVL exhibited minimal synergistic inhibitory activity (14.3%) when applied in combination with voriconazole (VRC). Antagonistic interactions were not observed. *In vivo* experiments conducted in *Galleria mellonella* revealed that EVL in combination with antifungal agents improved the larva survival rates in the ITC, VRC, POS, and AMB groups by 18.3%, 13.3%, 26.7%, and 13.3%, respectively. These data suggest that the combination treatment with antifungal agents and antifungal agents holds promise as a means of alleviating clinical aspergillosis.

## Introduction

*Aspergillus* species are major drivers of invasive fungal infections and associated mortality in immunosuppressed individuals (Kontoyiannis and Bodey, 2002). *Aspergillus* spp. spores are common in the environment and are thus easily transferred through the air such that they can cause cutaneous and respiratory infections (Thompson and Young, 2021). The widespread utilization of immunosuppressants, antibiotics, corticosteroids, and related drugs has contributed to the gradually rising rates of *Aspergillus* infections, particularly in susceptible individuals with chronic systemic diseases (Thompson and Young, 2021; Cadena et al., 2021). Invasive pulmonary aspergillosis has been identified as the most common cause of mortality among critically ill individuals (Ramirez and Garnacho-Montero, 2018), resulting in lung tissue inflammation, degradation, and necrosis together with the disruption of the pulmonary vasculature and consequent symptoms including asthma, hemoptysis, and respiratory failure. Management of this deadly disease remains challenging, but the development of novel antifungal azole drugs (e.g., voriconazole, posaconazole, and isavuconazole), amphotericin B (AMB) lipid formulations (liposomal AMB and AMB lipid complex), and echinocandins (e.g., caspofungin, micafungin, and anidulafungin) has greatly expanded the treatment options available to patients in recent years (Ledoux et al., 2020). Unfortunately, antifungal resistance remains an increasingly common finding in the context of aspergillosis, and the mortality rates remain high. In an effort to overcome the limitations of current therapeutic strategies, combination therapies must be explored through *in vitro* testing.

Target of rapamycin (TOR) is a highly conserved serine/threonine kinase that serves as a primary regulator of cellular metabolism, protein synthesis, and cell cycle progression in eukaryotes that is frequently deregulated in cancer (Baldin et al., 2015). As such, TOR is an attractive target for cancer therapy and has an important role in the antifungal area (Gao et al., 2016). Rapamycin is a macrolide that has antifungal and immunosuppressive effects, but its inherent toxicities limit its clinical application (Sehgal, 2003). Recently developed rapamycin analogs, such as INK128, have been proven to be less toxic and to synergistically interact with antifungal agents (Gao et al., 2016). TOR inhibitors additionally exhibit great potential for the antifungal treatment of cancer patients who are vulnerable to fungal infections, including those caused by *Aspergillus* species.

Everolimus (EVL) is a rapamycin derivative with good oral availability that has shown great promise as an agent capable of treating cancer and preventing acute rejection in solid organ transplant recipients in randomized clinical trials (Tedesco-Silva et al., 2022). As these patients frequently suffer from opportunistic pathogen infections including *Aspergillus* spp., this work was developed to explore the combinatory influence of EVL and AMB or azole antifungal agents in order to establish whether these compounds exhibit synergistic inhibitor activity against clinical *Aspergillus* isolates.

## Materials and Methods

### Fungal Strains

For this study, 35 clinical isolates were tested, which included 18 *Aspergillus fumigatus* isolates, 12 *Aspergillus flavus* isolates, and 5 *Aspergillus terreus* isolates, with testing being conducted *via* a 96-well plate-based approach, as detailed previously (Pierce et al., 2018). For quality control, *Candida parapsilosis* (ATCC22019) and *A. flavus* (ATCC204304) were used. All clinical isolates were obtained from patients suffering from invasive pulmonary aspergillosis and who had undergone molecular and morphological identification to confirm identity of the strain.

### Antifungal Agents

Antifungal drugs were purchased from Sigma Chemical Co. (St. Louis, MO, USA) in powder form, which included itraconazole (ITC; purity, ≥99%), voriconazole (VRC; purity, ≥99%), posaconazole (POS; purity, ≥99%), and AMB (purity, ≥80%). For the tested drugs, the working concentration range was 0.06–16 µg/ml. EVL (purity, ≥99%) was purchased from Selleck Chemicals (Houston, TX, USA), with a working concentration range of 0.25–32 µg/ml. All drugs were prepared following the broth microdilution method M38-A2 as detailed by the Clinical and Laboratory Standards Institute (CLSI) (Clinical and Laboratory Standards Institute, 2008).

### Inoculum Preparation

Conidia were suspended in sterile distilled water at 1–5 × 10^6^ colony forming units (CFU)/ml from cultures grown for 4 days on Sabouraud dextrose agar (SDA), followed by 100-fold dilution using RPMI-1640 to yield a final concentration of 1–5 × 10^4^ CFU/ml.

### *In Vitro* Antifungal Activity of Individual Tested Agents

The individual minimal inhibitory concentrations (MICs) of EVL, ITC, VRC, POS, and AMB were determined according to the M38-A2 method. Briefly, 100 μl of the prepared inoculum and 100 μl of serially diluted test drugs were added to the wells of 96-well plates. Following incubation for 48 h at 35°C, the MICs were established based on the lower drug concentration completely inhibiting growth. Assays were conducted in triplicate.

### Antimicrobial Checkerboard Synergy Assay

The interactive effects between EVL and the selected antimicrobial drugs on the target fungal strains were analyzed through a microdilution checkerboard approach. Briefly, serially diluted EVL (50 μl) was added horizontally to a 96-well plate, with 50 μl of serially diluted antifungal drugs of interest, then added in a vertical direction to the wells already containing 100 μl of the prepared inoculum following incubation for 48 h at 35°C. The interaction of EVL with antifungal agents was referred to as the fractional inhibitory concentration index (FICI) (Odds, 2003), which was classified as follows: FICI of ≤0.5, synergy; FICI from >0.5 to ≤4, no interaction (indifference); and FICI of >4, antagonism. All tests were performed in triplicate.

### *In Vivo* Analyses of the Combined Effects of EVL and Antifungal Agents Against *A. fumigatus*

A *Galleria mellonella* (300 mg; Sichuan, China) *A. fumigatus* infection model was used to conduct survival testing examining the *in vivo* impact of EVL treatment alone or in combination with antifungal agents *in vivo.* Prior to use, the larvae were kept at room temperature in the dark, while *A. fumigatus* AF1 was harvested using sterile plastic loops to gently scrape the culture surface following growth for 4 days on SDA. Fungi were rinsed two times and suspended in sterile saline at 1 × 10^8^ CFU/ml. The following nine intervention treatment groups were established: EVL treatment group, ITC treatment group, POS treatment group, VRC treatment group, AMB treatment group, EVL+ITC treatment group, EVL+POS treatment group, EVL+VRC treatment group, and EVL+AMB treatment group. Moreover, a sterile saline group, a conidia suspension group, and a no contact group were set as controls.

The conidia suspension (10 μl per larva, 1 × 10^8^ CFU/ml) and control solution or antifungal agents (1 μg per larvae, 200 mg/L) were injected using a Hamilton syringe (25-gauge, 50 μl) into the last left proleg. The survival rate of the larvae was recorded every day for 6 days after infection. The Kaplan–Meier method was used to analyze the survival curves, and the log-rank (Mantel–Cox) test was utilized to determine differences. At a *p*-value of <0.05, differences were considered significant.

## Results

### *In Vitro* Antifungal Activities of Individual Tested Agents

The MIC values of the tested drugs used to treat planktonic *Aspergillus* isolates were ≥32 μg/ml for EVL, 0.125–4 μg/ml for ITC, 0.125–4 μg/ml for VRC, 0.062–4 μg/ml for POS, and 1–16 μg/ml for AMB (**Table 1**).

**Table 1:** Results of the minimal inhibitory concentrations (MICs) and fractional inhibitory concentration indices (FICIs) with the combinations of everolimus (EVL) and antifungal agents against *Aspergillus* strains^a^.

Strains	MIC (μg/ml)					MIC [drug_A_/drug_B_ (μg/ml)] (FICI)			
	EVL	ITC	VRC	POS	AMB	EVL/ITC	EVL/VRC	EVL/POS	EVL/AMB
*Aspergillus fumigatus*									
AF1	>32	1	1	1	4	1/0.125 (0.15, S)	2/0.062 (0.12, S)	1/0.25 (0.28, S)	1/1 (0.28, S)
AF2	>32	4	0.5	2	4	2/0.5 (0.18, S)	0.25/0.5 (1.01, I)	1/0.5 (0.28, S)	0.25/4 (1.01, I)
AF3	>32	2	0.25	4	4	1/0.5 (0.28, S)	0.25/0.25 (1.01, I)	0.5/1 (0.26, S)	1/1 (0.28, S)
AF4	>32	4	4	0.25	2	1/1 (0.28, S)	0.25/2 (0.51, I)	0.25/0.25 (1.01, I)	2/0.5 (0.31, S)
AF5	>32	2	0.25	2	4	1/0.5 (0.28, S)	0.25/0.25 (1.01, I)	1/0.5 (0.28, S)	1/2 (0.53, I)
AF6	>32	2	0.5	2	8	2/0.5 (0.31, S)	0.25/0.5 (1.01, I)	1/0.5 (0.28, S)	1/2 (0.28, S)
AF7	>32	2	0.25	1	4	2/0.5 (0.31, S)	0.25/0.25 (1.01, I)	1/0.25 (0.28, S)	1/1 (0.28, S)
AF8	>32	1	2	0.062	4	4/0.25 (0.37, S)	2/0.5 (0.31, S)	0.5/0.062 (1.01, I)	0.5/1 (0.26, S)
AF9	>32	2	0.5	2	4	4/0.5 (0.37, S)	0.25/0.5 (1.01, I)	1/0.5 (0.28, S)	1/1 (0.28, S)
AF10	>32	0.25	0.25	2	8	4/0.062 (0.37, S)	0.25/0.25 (1.01, I)	2/0.5 (0.31, S)	1/2 (0.28, S)
AF11	>32	0.5	0.5	0.062	1	4/0.125 (0.37, S)	0.25/0.5 (1.01, I)	0.25/0.062 (1.01, I)	1/0.25 (0.28, S)
AF12	>32	0.25	0.25	4	4	4/0.062 (0.37, S)	0.25/0.25 (1.01, I)	1/1 (0.28, S)	2/1 (0.31, S)
AF13	>32	0.5	0.5	0.062	2	4/0.125 (0.37, S)	0.25/0.5 (1.01, I)	0.25/0.062 (1.01, I)	0.25/1 (0.51, I)
AF14	>32	0.5	0.5	0.5	4	4/0.125 (0.37, S)	4/0.25 (0.63, I)	1/0.062 (0.15, S)	0.5/2 (0.51, I)
AF15	>32	0.5	0.25	0.25	2	4/0.125 (0.37, S)	0.5/0.25 (1.01, I)	1/0.062 (0.28, S)	1/1 (0.53, I)
AF16	>32	0.25	0.5	0.125	4	8/0.062 (0.5, I)	0.25/0.5 (1.01, I)	0.25/0.062 (0.51, I)	0.5/1 (0.27, S)
AF17	>32	0.5	0.5	0.062	1	2/0.25 (0.56, I)	0.25/0.5 (1.01, I)	0.25/0.062 (1.01, I)	0.5/0.25 (0.27, S)
AF18	>32	0.5	0.5	0.062	4	2/0.25 (0.56, I)	0.25/0.5 (1.01, I)	0.25/0.062 (1.01, I)	2/1 (0.31, S)
*Aspergillus flavus *									
AFL1	>32	1	0.25	2	4	2/0.125 (0.19, S)	0.25/0.25 (1.01, I)	1/0.25 (0.16, S)	1/1 (0.28, S)
AFL2	>32	2	0.5	2	4	0.5/0.5 (0.27, S)	2/0.125 (0.31, S)	1/0.25 (0.16, S)	1/0.25 (0.09, S)
AFL3	>32	2	1	2	16	1/0.5 (0.28, S)	1/0.5 (0.53, I)	2/0.5 (0.31, S)	0.25/4 (0.25, S)
AFL4	>32	2	1	4	8	1/0.5 (0.28, S)	0.25/1 (1.01, I)	1/1 (0.28, S)	1/2 (0.28, S)
AFL5	>32	2	0.5	2	8	1/0.5 (0.28, S)	0.25/0.5 (1.01, I)	1/0.25 (0.16, S)	4/2 (0.375, S)
AFL6	>32	0.5	0.125	1	2	1/0.125 (0.28, S)	2/0.062 (0.56, I)	1/0.062 (0.09, S)	8/0.5 (0.5, I)
AFL7	>32	0.5	1	0.125	8	2/0.125 (0.31, S)	2/0.25 (0.31, S)	1/0.062 (0.53, I)	4/1 (0.25, S)
AFL8	>32	0.5	0.5	0.25	4	2/0.125 (0.31, S)	0.25/0.5 (1.01, I)	1/0.062 (0.28, S)	1/1 (0.28, S)
AFL9	>32	0.25	0.5	0.25	4	2/0.062 (0.31, S)	0.25/0.5 (1.01, I)	1/0.062 (0.28, S)	1/1 (0.28, S)
AFL10	>32	0.5	0.125	0.5	2	2/0.125 (0.31, S)	8/0.062 (0.52, I)	2/0.062 (0.18, S)	2/0.5 (0.31, S)
AFL11	>32	0.5	0.5	1	4	2/0.125 (0.31, S)	2/0.25 (0.56, I)	1/0.125 (0.16, S)	0.5/2 (0.52, I)
AFL12	>32	0.25	0.5	0.25	4	2/0.062 (0.31, S)	4/0.25 (0.62, I)	1/0.062 (0.28, S)	8/1 (0.5, I)
*Aspergillus terreus*									
AT1	>32	0.5	1	1	8	2/0.125 (0.31, S)	0.5/0.5 (0.52, I)	1/0.25 (0.28, S)	1/2 (0.28, S)
AT2	>32	2	0.5	4	4	4/0.5 (0.37, S)	0.25/0.5 (1.01, I)	0.5/1 (0.26, S)	1/2 (0.53, I)
AT3	>32	0.5	0.5	0.062	2	4/0.125 (0.37, S)	0.25/0.5 (1.01, I)	0.25/0.062 (1.01, I)	4/1 (0.63, I)
AT4	>32	0.125	0.5	2	8	0.25/0.062 (0.51, I)	2/0.125 (0.31, S)	1/0.25 (0.15, S)	1/2 (0.28, S)
AT5	>32	0.125	0.25	4	4	0.25/0.062 (0.51, I)	0.25/0.25 (1.01, I)	1/1 (0.28, S)	2/1 (0.31, S)

ITC, itraconazole; VRC, voriconazole; POS, posaconazole; AMB, amphotericin B; S, synergy (FICI ≤ 0.5); I, indifference; (no interaction, FICI from >0.5 to ≤4); EVL, everolimus.

^a^MICs were the concentrations that achieved 100% growth inhibition.

### *In Vitro* Interactions Between EVL and Antifungal Agents

Combining EVL and ITC reduced the MIC values to 0.25–8 and 0.062–1 μg/ml, respectively, consistent with the best synergistic interaction against the tested *Aspergillus *spp. (85.7%), including all *A. flavus* strains, 15 A*. fumigates* strains, and 3 A*. terreu*s strains (**Tables 1** and **2**).

**Table 2:** Summary of drug interactions for the combination of everolimus (EVL) and antifungal agents.

Species (*n*)	*n* (%) of isolates showing synergism for the combination			
	EVL+ITC	EVL+VRC	EVL+POS	EVL+AMB
*Aspergillus fumigatus* (18)	15 (83.3)	2 (11.1)	11 (61.1)	13 (72.2)
*Aspergillus flavus* (12)	12 (100)	2 (16.7)	11 (91.7)	9 (75)
*Aspergillus terreus* (5)	3 (60)	1 (20)	4 (80)	3 (60)
Total (35)	30 (85.7)	5 (14.3)	26 (74.2)	25 (71.4)

ITC, itraconazole; VRC, voriconazole; POS, posaconazole; AMB, amphotericin B; EVL, everolimus.

When EVL and POS were combined, the MIC values for these two compounds decreased to 0.25–2 and 0.062–1 μg/ml, respectively, exhibiting synergistic activity against 74.2% of the tested *Aspergillus* spp., including 11 A*. fumigates* strains, 11 A*. flavus* strains, and 4 A*. terreus* strains.

Combining EVL and AMB reduced the MIC values for these two compounds to 0.25–8 and 0.5–4 μg/ml, respectively, consistent with synergistic activity against *Aspergillus* spp. (71.4%), including 13 A*. fumigates* strains, 9 A*. flavus* strains, and 3 A*. terreus* strains.

Combining EVL and VRC resulted in respective effective working ranges of 0.25–8 and 0.5–4 μg/ml, with synergism being observed against five tested *Aspergillus* isolates, including 2 *A. fumigates* strains, 2 *A. flavus* strains, and 1 *A. terreus* strain.

No antagonistic interactions were observed between the tested drugs.

### *In Vivo* Interactions Between EVL and Azoles Antifungal Agents Against *A. fumigates*

**Figure 1:**
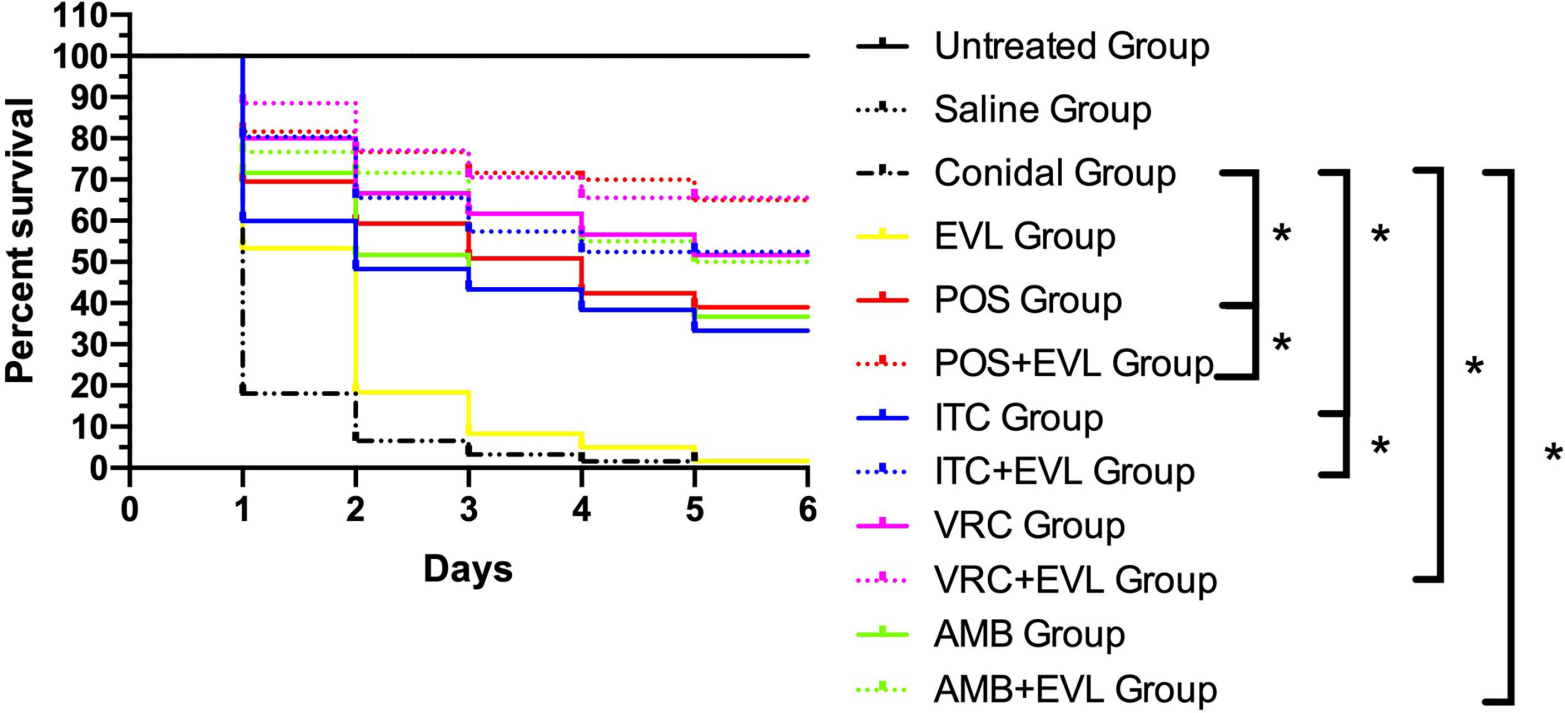
Survival curves for *Galleria mellonella* infected with *Aspergillus fumigates* AF1. *Untreated Group*, uninfected wild–type larvae, *Conidial Group*, larvae infected with *A. fumigates* without any treatment, *Saline Group*, wild–type larvae injected with saline, *EVL Group*, *A. fumigates*–infected larvae treated with everolimus (EVL) only, *ITC Group*, *A. fumigates*–infected larvae treated with itraconazole (ITC) only, *VRC Group*, *A. fumigates*–infected larvae treated with voriconazole (VRC) only, *POS Group*, *A. fumigates* infected larvae treated with posaconazole (POS) only, *AMB Group*, *A. fumigates*–infected larvae treated with amphotericin B (AMB) only, *EVL+ITC Group*, *A. fumigates*–infected larvae treated with EVL combined with ITC, *EVL+VRC Group*, *A. fumigates*–infected larvae treated with EVL combined with VRC, *EVL+POS Group*, *A. fumigates*–infected larvae treated with EVL combined with POS, *EVL+AMB Group*, *A. fumigates*–infected larvae treated with EVL combined with AMB.*p < 0.05.

The larval survival rates in the groups after treatments with ITC, VRC, POS, and AMB alone were 33.3%, 51.67%, 38.33%, and 36.67%, respectively. When combined with EVL, the survival rates in the EVL+ITC, EVL+VRC, EVL+POS, and EVL+AMB groups increased to 51.67%, 65%, 65%, and 50%, respectively. Treatment with EVL combined with antifungal agents significantly (*p* < 0.05) enhanced the survival of *A. fumigates*-infected larvae, especially in the EVL+POS and EVL+ITC groups, with respective survival rate increases of 26.7% and 18.3% (**Figure 1**). Treatment with EVL alone had no impact on the outcomes of *A. fumigates* infection.

## Discussion

Compounds that can inhibit the mammalian target of rapamycin (mTOR) signaling pathway are frequently utilized to treat patients with cancer and individuals who have undergone organ transplantation. However, these drugs are also immunosuppressive and can increase the risk of invasive fungal infections, such as invasive aspergillosis, thus necessitating simultaneous antifungal treatment. However, when patients undergo antifungal treatment, they may exhibit more complications, such as secondary infections, organ failure, or death (Vidanapathirana et al., 2021). If immunosuppressive agents can exhibit intrinsic antifungal activity or enhance the antifungal ability of antifungals, they may facilitate better outcomes for patients.

Recent studies have suggested that the TOR signaling pathway may be a potential regulatory node in *A. fumigates* (Crespo and Hall, 2002), as TOR is a known regulator in stress response, nutrient sensing, cell cycle progression, degradation process, and protein biosynthesis [Baldin et al., 2015). The TOR inhibitor rapamycin was originally identified as being able to suppress the growth of many species of fungi, including *Candida, Aspergillus*, *Fusarium*, *Cryptococcus*, and *Penicillium*, as well as dermatophyte species [Rohde and Cardenas, 2004; Tedesco-Silva et al., 2022). Rapamycin and caspofungin have also been shown to positively interact when used to treat *A. fumigatus* isolates [Kontoyiannis et al., 2003), while the analogous drug INK128 synergized with azoles in the treatment of a range of *Aspergillus* spp (Gao et al., 2016).. In clinical settings, however, rapamycin was found to be a poor antifungal agent owing to its potent immunosuppressive activity. Although promising, INK128 is still undergoing clinical evaluation through appropriate drug trials (Li et al., 2021).

In this study, we investigated another TOR inhibitor, EVL, which can be administered orally and has received approval from the United States Food and Drug Administration (FDA) for prophylactic treatment (Gabardi and Baroletti, 2010; Hasskarl, 2018). In the context of transplantation, EVL exhibits immunosuppressive properties and can prevent acute organ rejection. Moreover, it may be sufficiently potent to enable the minimization or elimination of calcineurin inhibitors when managing patients who have received kidney transplants. In oncological settings, EVL can effectively treat individuals with renal cell carcinoma that is resistant to all forms of treatment. In cardiological contexts, EVL is available in the form of a drug–coated stent that is used in percutaneous coronary interventions to prevent restenosis (Rodríguez-Arias et al., 2020). In patients with renal cell carcinoma and transplant recipients, EVL appears to have an extensive profile of adverse reactions. Currently, the oral EVL dose for cardiac and renal transplant recipients is 0.75 mg, twice daily (Dunn and Croom, 2006), whereas it is administered once per day at a 10–mg dose in oncological contexts (Oudard et al., 2009).

In this study, a single–agent EVL treatment failed to exhibit any activity against the tested *Aspergillus* spp. even at the highest dosage. When combined with antifungal agents, however, EVL exhibited synergistic activity, inhibiting the growth of *A. fumigates*, *A. flavus*, and *A. terreus* isolates. No antagonistic interactions between these different antifungal agents were detected. For *in vivo* experiments, *G. mellonella* was used as an animal model for *A. fumigates* infection. When combined with EVL, the survival of larvae in the ITC, VRC, POS, and AMB groups respectively increased by 18.3%, 13.3%, 26.7%, and 13.3%, indicating that these combinations, and particularly EVL+POS, are promising treatments for clinical *Aspergillus* infections.

Although further work is needed to clarify the underlying mechanistic basis for the observed synergy, these data highlight a promising therapeutic option to alleviate clinical *Aspergillus* infections, particularly in cancer patients and individuals undergoing organ transplantation, with the synergistic combinations of these drugs being recommended as a means of achieving better outcomes.

## Conclusions

In summary, we herein found that the TOR inhibitor EVL exhibits synergistic antifungal activity with azoles and AMB when used to treat *Aspergillus* spp., indicating that the combinations of these pharmaceutical agents may be a more reliable therapeutic option for the treatment of patients with aspergillosis.

## Data Availability Statement

The original contributions presented in the study are included in the article. Further inquiries can be directed to the corresponding author.

## Author Contributions

HJ and JX carried out the *in vitro* antifungal experiment. LT and PJ collected and analyzed the experiment data. YS and JT designed, interpreted the experiment data, and wrote the manuscript. LY revised the manuscript critically for important content. All authors contributed to the article and approved the submitted version.

## Funding

This work was supported by the National Natural Science Foundation of China (grant no. 82102418 to JT, grant no. 82173429 to LY), Health Commission of Hubei Province Scientific Research Project (grant no. WJ2021M261 to YS), the Natural Science Foundation of Hubei Province (grant no. 2019CFB567 to YS), Shanghai Municipal Commission of Health and Family Planning (grant no. 201940476 to LY), and the Science and Technology Commission of Shanghai Municipality (grant no. 21Y11904900 to LY). The funders had no role in the study design, data analysis, the decision to publish, or the preparation of the manuscript.

## Conflict of Interest

The authors declare that the research was conducted in the absence of any commercial or financial relationships that could be construed as a potential conflict of interest.

## Publisher’s Note

All claims expressed in this article are solely those of the authors and do not necessarily represent those of their affiliated organizations, or those of the publisher, the editors and the reviewers. Any product that may be evaluated in this article, or claim that may be made by its manufacturer, is not guaranteed or endorsed by the publisher.

## References

[B1] Baldin C. Valiante V. Kruger T. Schafferer L. Haas H. Kniemeyer O.. (2015). Comparative Proteomics of a Tor Inducible *Aspergillus Fumigatus* Mutant Reveals Involvement of the Tor Kinase in Iron Regulation. Proteomics 15, 2230–2243. doi: 10.1002/pmic.201400584 25728394

[B2] Cadena J. Thompson G. R. 3rd Patterson T. F. (2021). Aspergillosis: Epidemiology, Diagnosis, and Treatment. Infect. Dis. Clin. North Am. 35, 415–434. doi: 10.1016/j.idc.2021.03.008 34016284

[B3] Clinical and Laboratory Standards Institute (2008). Reference Method for Broth Dilution Antifungal Susceptibility Testing of Filamentous Fungi. Approved Standard, 2nd Ed CLSI Document M38-A2 (Wayne, PA: Clinical and Laboratory Standards Institute).

[B4] Crespo J. L. Hall M. N. (2002). Elucidating TOR Signaling and Rapamycin Action: Lessons From Saccharomyces Cerevisiae. Microbiol. Mol. Biol. Rev. 66, 579–591. doi: 10.1128/mmbr.66.4.579-591.2002 12456783 PMC134654

[B5] Dunn C. Croom K. F. (2006). Everolimus: A Review of its Use in Renal and Cardiac Transplantation. Drugs 66, 547–570. doi: 10.2165/00003495-200666040-00009 16597167

[B6] Gabardi S. Baroletti S. A. (2010). Everolimus: A Proliferation Signal Inhibitor With Clinical Applications in Organ Transplantation, Oncology, and Cardiology. Pharmacotherapy 30, 1044–1056. doi: 10.1592/phco.30.10.1044 20874042

[B7] Gao L. Ding X. Liu Z. Wu Q. Zeng T. Sun Y. (2016). *In Vitro* Interactions Between Target of Rapamycin Kinase Inhibitor and Antifungal Agents Against *Aspergillus* Species. Antimicrob. Agents Chemother. 60, 3813–3816. doi: 10.1128/AAC.02921-15 26976874 PMC4879397

[B8] Gao L. Sun Y. He C. Li M. Zeng T. Lu Q. (2016). INK128 Exhibits Synergy With Azoles Against *Exophiala* Spp. And Fusarium Spp. Front. Microbiol. 7. doi: 10.3389/fmicb.2016.01658 PMC5071350 27812353

[B9] Hasskarl J. (2018). Everolimus. Recent Results Cancer Res. 211, 101–123. doi: 10.1007/978-3-319-91442-8_8 30069763

[B10] Kontoyiannis D. P. Bodey G. P. (2002). Invasive Aspergillosis in 2002: An Update. Eur. J. Clin. Microbiol. Infect. Dis. 21, 161–172. doi: 10.1007/s10096-002-0699-z 11957017

[B11] Kontoyiannis D. P. Lewis R. E. Osherov N. Albert N. D. May G. S. (2003). Combination of Caspofungin With Inhibitors of the Calcineurin Pathway Attenuates Growth *In Vitro* in *Aspergillus* Species. J. Antimicrob. Chemother. 51, 313–316. doi: 10.1093/jac/dkg090 12562696

[B12] Ledoux M. P. Guffroy B. Nivoix Y. Simand C. Herbrecht R. (2020). Invasive Pulmonary Aspergillosis. Semin. Respir. Crit. Care Med. 41, 80–98. doi: 10.1055/s-0039-3401990 32000286

[B13] Li Y. Xu Y. Liu X. Yan X. Lin Y. Tan Q.. (2021). mTOR Inhibitor INK128 Promotes Wound Healing by Regulating MDSCs. Stem Cell Res. Ther. 12, 170. doi: 10.1186/s13287-021-02206-y 33691762 PMC7944919

[B14] Odds F. C. (2003). Synergy, Antagonism, and What the Chequerboard Puts Between Them. J. Antimicrob. Chemother. 52, 1. doi: 10.1093/jac/dkg301 12805255

[B15] Oudard S. Medioni J. Aylllon J. Barrascourt E. Elaidi R. T. Balcaceres J.. (2009). Everolimus (RAD001): An mTOR Inhibitor for the Treatment of Metastatic Renal Cell Carcinoma. Expert Rev. Anticancer Ther. 9, 705–717. doi: 10.1586/era.09.27 19496707

[B16] Pierce C. G. Uppuluri P. Tristan A. R. Wormley F. J. Mowat E. Ramage G.. (2018). A Simple and Reproducible 96-Well Plate-Based Method for the Formation of Fungal Biofilms and its Application to Antifungal Susceptibility Testing. Nat. Protoc. 3, 1494–1500. doi: 10.1038/nport.2008.141 PMC2741160 18772877

[B17] Ramirez P. Garnacho-Montero J. (2018). Invasive Aspergillosis in Critically Ill Patients. Rev. Iberoam Micol 35, 210–216. doi: 10.1016/j.riam.2018.07.001 30554674

[B18] Rodríguez-Arias J. J. Ortega-Paz L. Brugaletta S. (2020). Durable Polymer Everolimus-Eluting Stents: History, Current Status and Future Prospects. Expert Rev. Med. Devices 17, 671–682. doi: 10.1080/17434440.2020.1784005 32543934

[B19] Rohde J. R. Cardenas M. E. (2004). Nutrient Signaling Through TOR Kinases Controls Gene Expression and Cellular Differentiation in Fungi. Curr. Top. Microbiol. Immunol. 279, 53–72. doi: 10.1007/978-3-642-18930-2_4 14560951

[B20] Sehgal S. N. (2003). Sirolimus: its discovery, biological properties, and mechanism of action. Transplant. Proc. 35 (3 Suppl), 7S–14S. doi: 10.1016/s0041-1345(03)00211-2 12742462

[B21] Tedesco-Silva H. Saliba F. Barten M. J. De Simone P. Potena L. Gottlieb J.. (2022). An Overview of the Efficacy and Safety of Everolimus in Adult Solid Organ Transplant Recipients. Transplant. Rev. (Orlando) 36, 100655. doi: 10.1016/j.trre.2021.100655 34696930

[B22] Thompson G. R. 3rd Young J. H. (2021). Aspergillus Infections. N Engl. J. Med. 385, 1496–1509. doi: 10.1056/NEJMra2027424 34644473

[B23] Vidanapathirana M. Minuvanpitiya G. Karunaratne R. Fernando A. (2021). Triple Infection With Disseminated Tuberculosis, Invasive Aspergillosis and COVID-19 in an Organ Transplant Recipient With Iatrogenic Immunosuppression. BMJ Case Rep. 14, e245131. doi: 10.1136/bcr-2021-245131 PMC8356183 34376424

